# Effect of Topical Application of Povidone-Iodine on the Number of Bacteria Entering the Lower Respiratory Tract of Intubated Patients: A Randomized Controlled Trial

**DOI:** 10.7759/cureus.91274

**Published:** 2025-08-30

**Authors:** Yuki Sakamoto, Arisa Tanabe, Yuri Uchida, Makiko Moriyama, Madoka Funahara, Akira Imakiire, Sakiko Soutome, Yuka Kojima

**Affiliations:** 1 Oral Surgery, Kansai Medical University Medical Center, Moriguchi, JPN; 2 Oral Health Sciences, Kyushu Dental University, Kitakyusyu, JPN; 3 Oral Health, Nagasaki University Graduate School of Biomedical Sciences, Nagasaki, JPN; 4 Dentistry and Oral Surgery, Kansai Medical University Hospital, Hirakata, JPN; 5 Dentistry and Oral Surgery, Kansai Medical University, Hirakata, JPN

**Keywords:** intubation, pneumonia, povidone-iodine, toothbrushing, ventilator

## Abstract

Objective: The aim of the study was to evaluate and compare the efficacy of povidone-iodine (PI) oral care and toothbrushing in reducing the number of viable bacteria in subglottic secretions of intubated patients. Methods: This open-label randomized controlled trial included 20 ICU patients undergoing mechanical ventilation. Patients were assigned to either the PI or the toothbrushing groups. Subglottic secretions were collected immediately before oral care (baseline) and at 1, 2, 3, and 6 hours after the intervention. Viable bacterial counts were quantified using delayed real-time polymerase chain reaction. The Mann-Whitney U test was used for statistical comparison. Results: The PI group showed significant reductions in bacterial counts at 2, 3, and 6 hours after care (p=0.023, 0.002, and <0.001, respectively), whereas the toothbrushing group did not show significant changes. Conclusion: PI oral care effectively reduces viable bacterial load in the lower respiratory tract and may serve as a safe and cost-effective alternative to chlorhexidine for ventilator-associated pneumonia prevention in Japan.

## Introduction

Ventilator-associated pneumonia (VAP) is a serious and common complication encountered in intensive care units (ICUs), posing a significant threat to the health of patients undergoing endotracheal intubation [1,2]. VAP is a type of hospital-acquired pneumonia that develops ≥48 hours after endotracheal intubation [3-5]. Although the reported incidence of VAP varies, it is estimated to occur in 5%-20% of patients receiving mechanical ventilation [6-8], with a higher risk observed in cases of prolonged intubation. The onset of VAP is associated with extended hospital stays, increased medical costs, greater use of antibiotics, and a mortality rate that can reach 20%-50% [9-11].

Endotracheal intubation impairs swallowing function and disrupts the self-cleaning mechanisms of the oral cavity, leading to a more than 100-fold increase in oral bacterial load [12,13]. The aspiration of saliva containing these increased oral bacteria into the lower respiratory tract is one of the major causes of VAP. As a specific intervention for the prevention of VAP, the "ventilator bundle" proposed by the US Institute for Healthcare Improvement (IHI) has been widely adopted [14,15]. Within this bundle, the use of chlorhexidine (CHX) gluconate, as part of oral care, is recommended, and many healthcare facilities conduct oral care using 0.12%-0.2% CHX mouthwash at least twice daily [15-17]. However, due to reported cases of anaphylactic shock associated with CHX in Japan, the use of this agent on the oral mucosa is prohibited [18].

In Japan, mouthwashes containing more than 0.05% CHX are not approved, and products with a concentration of 0.12% cannot be used [19]. Consequently, oral care in Japan predominantly involves brushing (Br). The "Oral Care Practice Guidelines for Intubated Patients," developed by the Japanese Society of Critical Care Nursing in 2021, also recommends Br-centered oral care along with moisturizing. However, Funahara et al. have reported that Br may temporarily cause the spread of oral bacteria into the airway [20,21]. The Japanese Society of Intensive Care Medicine guidelines [22] do not include specific recommendations for oral care in these patients.

In Japan, public health insurance began covering "perioperative oral care (POC)" in 2012, initially to prevent complications in patients undergoing cancer treatment. Since then, the scope of POC has expanded to include patients with stroke, joint replacement surgery, palliative care, and those undergoing surgery under general anesthesia. In 2024, coverage was further extended to include patients undergoing endotracheal intubation in ICUs. However, as mentioned above, 0.12% CHX cannot be used in Japan, and as a result, oral care using toothbrushes is commonly practiced in many facilities. A survey conducted by the Japanese Society of Oral Care across 302 hospitals with ICUs revealed that 92.4% of institutions performed oral care for intubated patients using toothbrushes in combination with sponge brushes and suction devices. However, numerous studies have shown that toothbrushing has no preventive effect on VAP [23-25] and, in fact, Br may disperse plaque-derived bacteria throughout the oral cavity [26], potentially increasing the risk of VAP. Therefore, we believe that Br is not an appropriate method of oral care for intubated patients.

Povidone-iodine (PI) exhibits broad-spectrum antimicrobial activity and has long been used in Japan for various oral disinfection purposes, such as wound cleaning and gargling. Additionally, it is inexpensive due to its coverage under the national health insurance system and could potentially serve as an alternative to 0.12% CHX for preventing VAP [27]. However, few studies have investigated the relationship between oral application of PI and the prevention of VAP. In a systematic review by Labeau [28], the effectiveness of PI for VAP prevention remained inconclusive.

This preliminary study aimed to evaluate the efficacy of PI in oral care for intubated patients by conducting a randomized comparison with the currently common practice of toothbrushing. The primary endpoint was the total bacterial count reaching the lower respiratory tract.

## Materials and methods

Study design

An open-label randomized controlled trial was conducted.

Participants

Inclusion Criteria

The study included patients who underwent endotracheal intubation management at Kansai Medical University Medical Center between November 2023 and October 2024, who were provided with an explanation of the study, and who gave their consent to participate. A total of 20 patients were targeted based on feasibility considerations. As this sample size was a preliminary study, no statistical sample size calculation was performed.

Exclusion Criteria

Patients with an allergy to iodine, edentulous patients, and those with minimal aspiration into the lower airway from whom subglottic secretions could not be collected were excluded.

Allocation and intervention

Patients were randomly assigned in a 1:1 ratio using computer software to either the Br group or the PI group.

In the Br group, toothbrushing using a standard soft-bristle toothbrush without toothpaste was performed for approximately two minutes while simultaneously using suction and wiping with a sponge brush. All Br procedures were performed by licensed dental hygienists who had been trained in the study protocol. In the PI group, the oral mucosa, including the tongue and buccal mucosa, was wiped using a sponge brush soaked in about 5 mL of 1.0% PI. While a 0.1% concentration of PI has been shown to be effective [29], we employed a 1% solution in this study, considering the potential dilution by saliva and the inactivation by organic substances. In the ICU, nurses perform oral suctioning, wiping, and aspiration of secretions above the endotracheal cuff every 6 hours. Since the present study observed patients for only 6 hours after oral care, the effects of routine nursing oral care did not influence the results.

Sample collection and measurement of bacteria

Subglottic secretions were aspirated and collected from the side port above the endotracheal tube cuff before oral care and at 1, 2, 3, and 6 hours after oral care. Samples were collected from subglottic secretions above the endotracheal tube cuff. This approach was chosen because direct sampling from the lower respiratory tract (e.g., bronchoalveolar lavage) would impose additional risks and burdens on critically ill, intubated patients, whereas subglottic aspiration can be performed safely through the endotracheal tube side port without additional invasiveness. The subglottic space also represents a clinically relevant reservoir where oral secretions accumulate before passing into the lungs, serving as a surrogate for assessing bacterial entry into the lower respiratory tract. This sample contained bacteria killed by PI. Therefore, we used the delayed real-time polymerase chain reaction (PCR) method [30], a newly developed method, to selectively and quantitatively analyze only viable bacteria.

The principle of this method is that a sample containing a mixture of viable and dead bacteria is first incubated in brain heart infusion (BHI) liquid medium for 4 hours to allow only viable bacteria to grow. Subsequently, a mixed samples of viable and dead bacteria (10:0, 5:5, 1:9, and 0:10 viable bacteria:dead bacteria) were prepared. The samples were first incubated in BHI liquid medium for 4 hours to allow only viable bacteria to grow, and then real-time PCR was performed. In real-time PCR, universal primers targeting 16 ribosomal RNA (forward: 5'-TCCTACGGGAGGCAGCAGT-3', reverse: 5'-GGACTACCAGGGTATCTAATCCTGTT-3') were used, as reported previously [31]. A standard line was prepared with the vertical axis representing the growth rate and the horizontal axis representing the logarithm of the percentage of viable bacteria. Next, the samples collected from patients were similarly subjected to 4 hours of liquid culture, followed by real-time PCR. The obtained values were then applied to the standard curve to quantify the number of viable bacteria.

Statistical analyses

The statistical analyses were performed using SPSS software (IBM Japan Ltd., Tokyo, Japan). The difference in the number of bacteria before and after oral care was analyzed using the Mann-Whitney U test. A p-value of <0.05 was considered statistically significant.

Ethics and registration

This study conformed to the tenets of the Declaration of Helsinki. Ethical approval was obtained from the Institutional Review Board (IRB) of Kansai Medical University Medical Center (2023041). Written informed consent was obtained from all patients. This study was registered with the University Hospital Medical Information Network Clinical Trials Registry (UMIN-CTR) (UMIN000057899).

## Results

Patient characteristics

**Figure 1:**
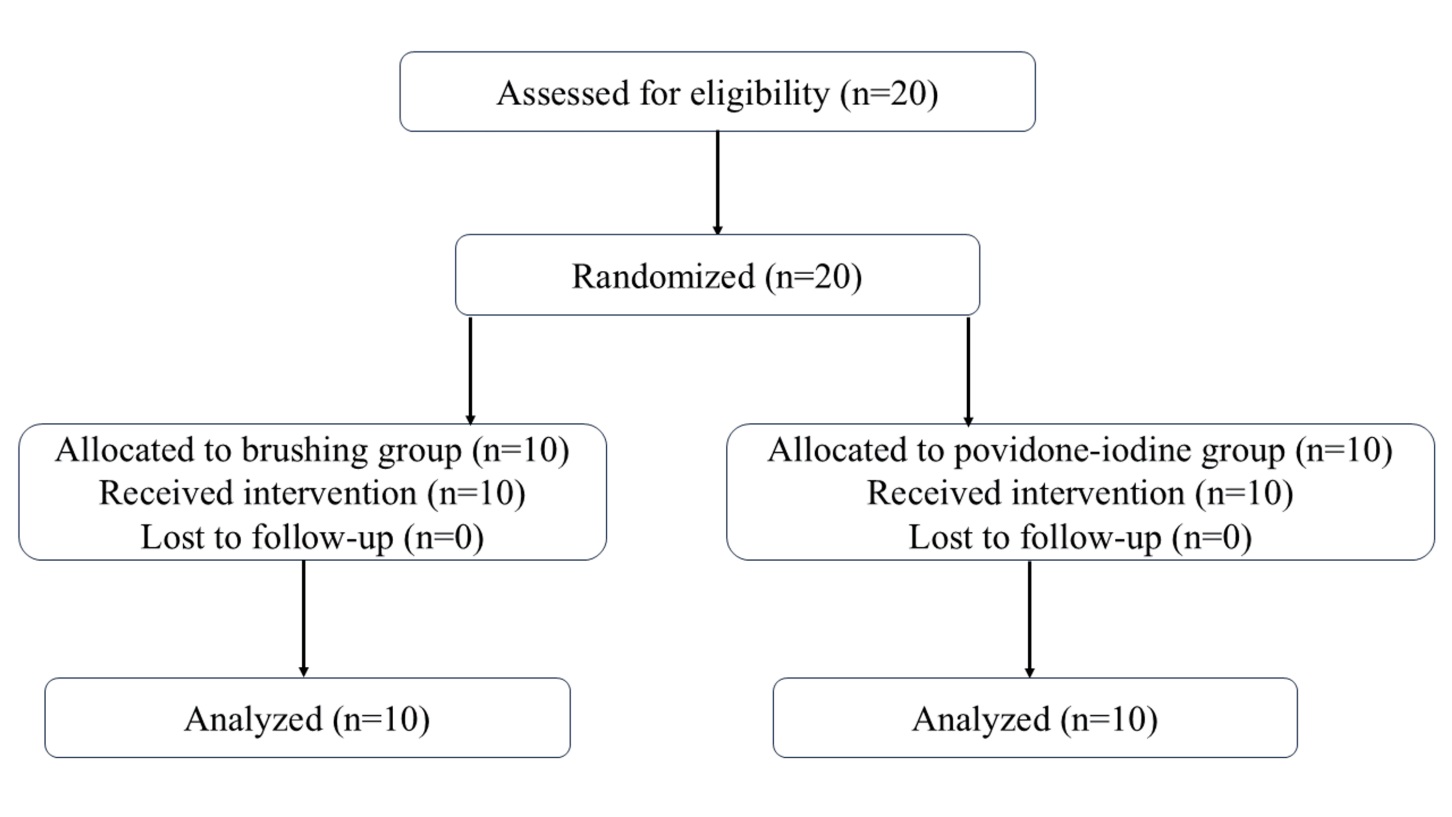
Consort flowchart diagram

The flow of patient enrollment, randomization, allocation, follow-up, and analysis is shown in Figure 1.

This study included 15 male and 5 female patients, with a mean age of 68.9 years. The most common primary diagnosis leading to ICU admission was cerebral hemorrhage (eight patients), followed by cervical vertebra fracture (three patients), pneumonia (two patients), and malignant tumor (two patients). Myocardial infarction, post-resuscitation encephalopathy, neurological disease, severe infectious disease, and acute leukemia accounted for one patient each. Blood test results are shown in Table 1, and no clinically significant abnormalities were observed in either group. 

**Table 1:** Patient characteristics SD: Standard deviation

Variable	Category	Number of patients/mean ± SD
Sex	Man	15
	Woman	5
Age		68.9±19.5
Primary disease	Cerebral hemorrhage	8
	Cervical vertebra fracture	3
	Pneumonia	2
	Malignant tumor	2
	Others	5
Hemoglobin	g/dL	10.0±1.75
Leukocytes	×10³/μL	9195±3346
Lymphocytes	×10³/μL	1521±793
Albumin	g/dL	2.49±0.504
Creatinine	mg/dL	1.15±1.59
Total number of patients		20

Change in bacterial count in supra-cuff secretions in the Br group

**Figure 2:**
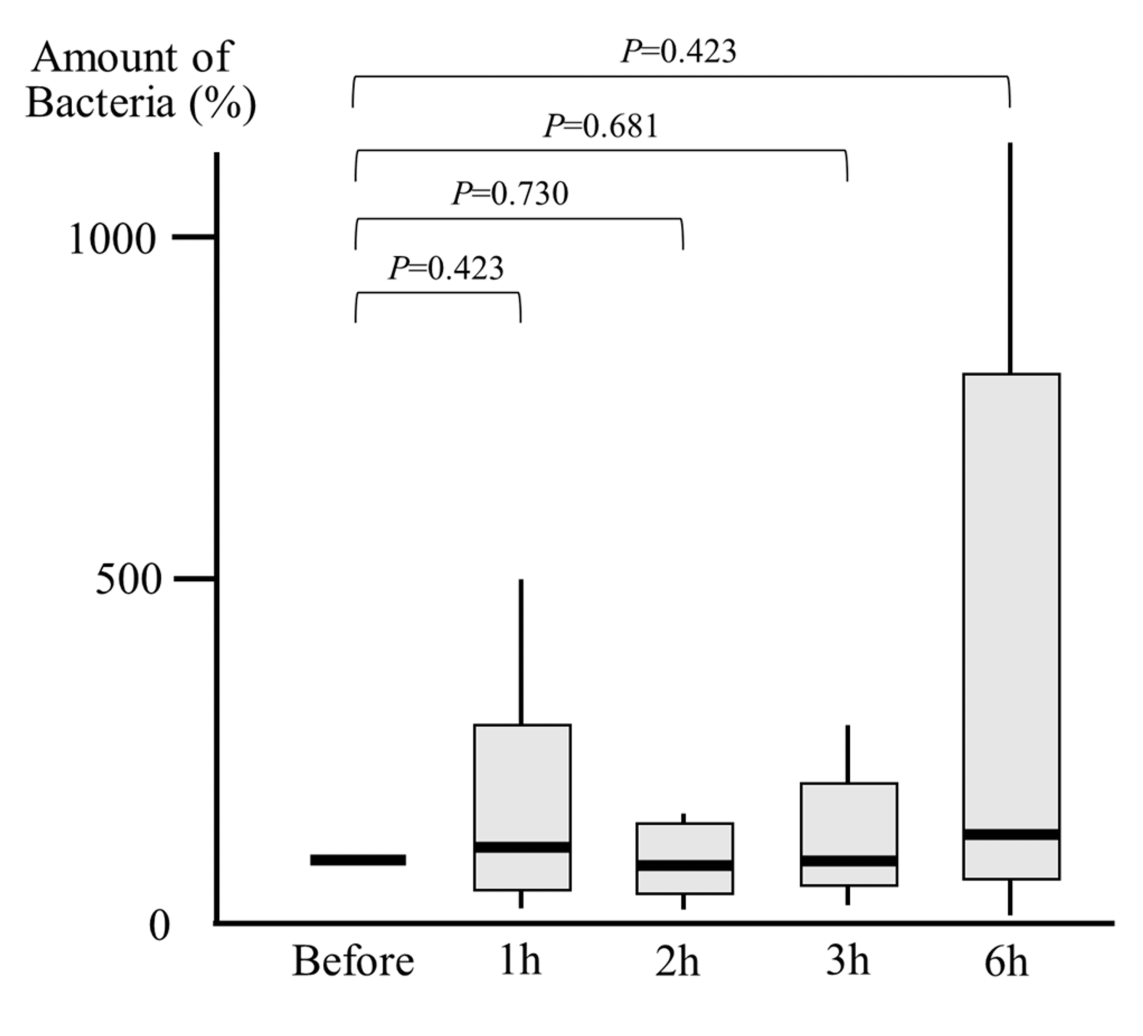
Bacteria count in supra-cuff secretions in the brushing (Br) group In the Br group, no significant change in bacterial counts was observed before and after oral care. Statistical analysis was performed using the Mann-Whitney U test.

When the bacterial count before oral care was set as 100% (log-transformed), the median bacterial counts at 1, 2, 3, and 6 hours after oral care were 119%, 93%, 97%, and 140%, respectively. None of these showed a statistically significant difference compared to the pre-oral care values (Figure 2).

Change in bacterial count in supra-cuff secretions in the PI group

**Figure 3:**
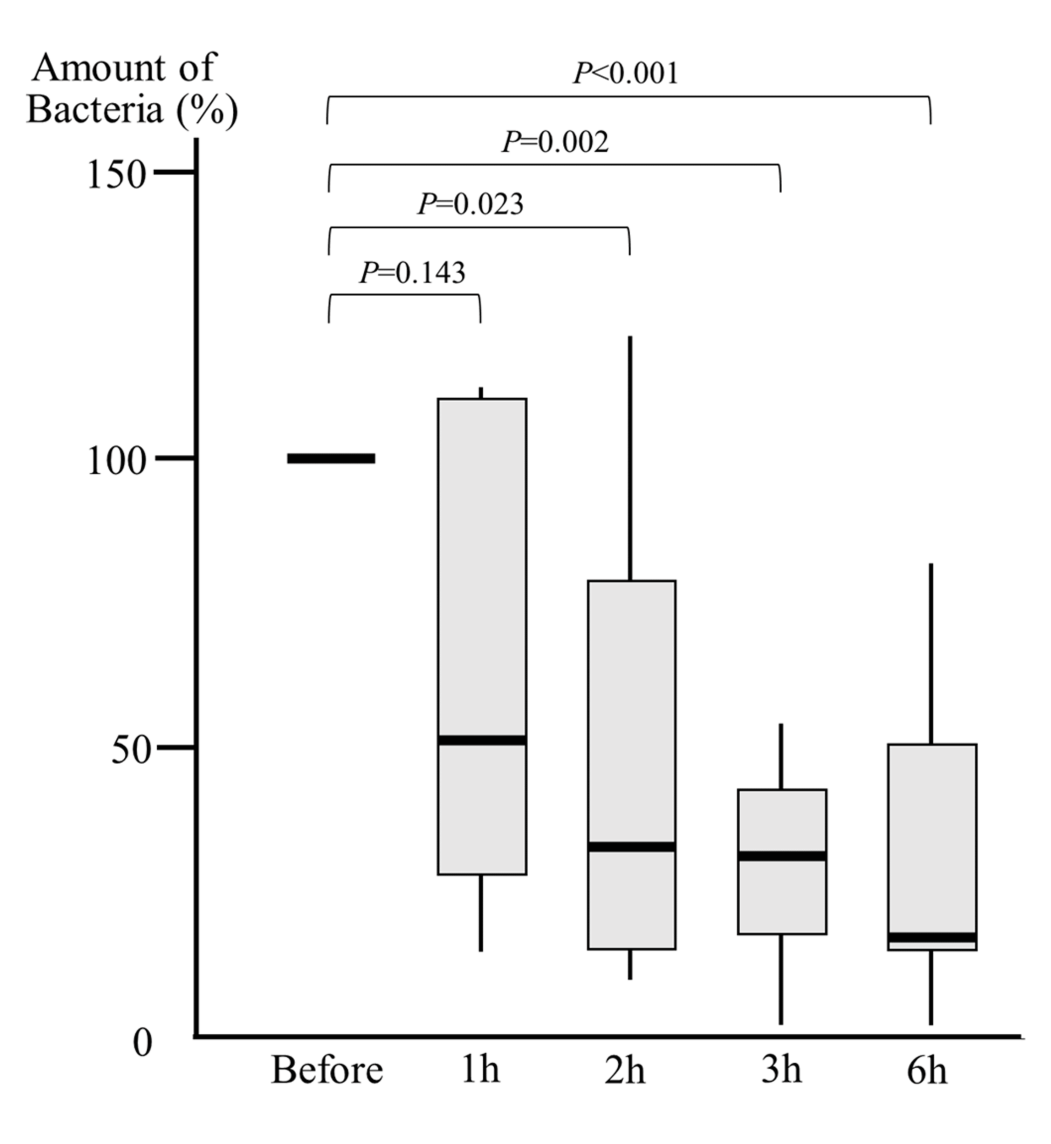
Bacteria count in supra-cuff secretions in the povidone-iodine (PI) group In the PI group, bacterial counts decreased after oral care, showing a significant reduction compared to pre-care levels at 2, 3, and 6 hours. Statistical analysis was performed using the Mann-Whitney U test.

When the bacterial count on the subglottic secretions after PI application was expressed as a percentage of the pre-application level (set at 100%, log-transformed), the median bacterial counts decreased to 50%, 33%, 30%, and 14% at 1, 2, 3, and 6 hours, respectively. Although the decrease at 1 hour was not statistically significant (p=0.143), significant reductions were observed at 2 hours (p=0.023), 3 hours (p=0.002), and 6 hours (p<0.001) (Figure 3).

## Discussion

This study revealed that oral care using PI significantly reduces the number of bacteria in secretions accumulating on the tracheal cannula of intubated patients, whereas Br alone does not have this effect. 

In other countries, the application of CHX to the oral cavity has been reported to be effective in preventing VAP [16]. However, in Japan, the Pharmaceutical and Medical Device Act restricts the use of CHX formulations with concentrations of ≥0.12% for oral application, limiting their use in clinical practice. In this context, PI, classified as an intermediate-level disinfectant, is permitted for use on the human body and possesses a broad antimicrobial spectrum. Therefore, simple interventions using the PI application may be effective for short-term intubated patients. Although CHX, classified as a low-level disinfectant, is effective against oral bacteria, some strains of *Pseudomonas aeruginosa*, a major causative agent of VAP, have been reported to exhibit resistance to CHX [32].

Iodine-based disinfectants, such as PI, are safe and effective for skin and mucosal disinfection and therefore represent a promising candidate for broader application in oral care. Supporting this, the Centers for Disease Control and Prevention (CDC) guidelines recommend intraoperative wound irrigation with iodophor solutions [33], and randomized controlled trials in spinal surgery have demonstrated that irrigation with 0.35% PI significantly reduces the incidence of deep surgical site infections. In addition, a meta-analysis of clean-contaminated and contaminated surgeries has shown that pre-closure irrigation with iodophor solution lowers the risk of superficial infections [34,35]. These findings collectively indicate that iodine-based disinfectants are both safe and effective, suggesting their potential usefulness in the field of oral care for intubated patients.

In a meta-analysis by Labeau et al. [28], oral antiseptics overall were shown to significantly reduce the incidence of VAP (risk ratio (RR): 0.67, 95% confidence interval (CI): 0.50-0.88, p=0.004), with a particularly significant effect observed in the CHX group (RR: 0.72, 95% CI: 0.55-0.94, p=0.02). In contrast, for PI, the point estimate suggested a roughly 60% risk reduction, but this was not statistically significant, likely due to the limited number of PI-related studies (only two), resulting in considerable uncertainty in the estimate (RR: 0.39, 95% CI: 0.11-1.36, p=0.14). To supplement this, Emami Zeydi reported that there was no statistically significant difference in the incidence of VAP between the PI oral care group and the placebo group (RR: 1.11, 95% CI: 0.67-1.82, p=0.69) [36]. 

The summaries of the two studies suggesting the efficacy of PI are as follows. Chua et al. [37] applied 1% PI three times daily along with daily toothbrushing (n=22), and Seguin et al. [38] used 10% PI six times daily (n=36) [28]. In the study by Chua et al. [37], the combination of Br and PI might have attenuated the observed antimicrobial effect, given that Br can temporarily disperse plaque-derived bacteria into the airway. This aligns with our findings in the Br group, where bacterial counts tended to increase transiently after toothbrushing. Notably, our study demonstrated a significant reduction in bacterial counts using only 1% PI once daily, suggesting that even lower concentrations and frequencies than those reported in previous studies can effectively suppress bacterial colonization above the endotracheal cuff. These results support the potential for 1% PI to serve as a feasible and efficient oral care strategy for intubated patients in Japanese clinical settings. Furthermore, Tsuda et al. demonstrated that oral application of 10% PI significantly reduced the bacterial count in the saliva of intubated patients, suggesting its potential usefulness [39]. A recent study by Imakiire et al. reported that applying PI in the oral cavity of tracheostomized patients with oral cancer led to a decrease in the number of bacteria reaching the lower respiratory tract [40].

In addition, in the Br group, although the differences were not statistically significant, bacterial counts tended to increase at 1 and 6 hours after oral care. This trend may reflect the possibility that toothbrushing disperses plaque-derived bacteria throughout the oral cavity, resulting in a temporary elevation of bacterial load in secretions above the endotracheal cuff. Such a phenomenon has also been suggested in previous reports [20], supporting the notion that Br might not be the most appropriate method of oral care for intubated patients.

In contrast, in Japanese clinical settings, concerns have been raised that PI mouthwashes may cause irritation or adverse effects on the oral mucosa or lead to oral dryness due to the presence of alcohol. However, such reports are largely based on expert opinion and anecdotal experience. The CDC guidelines [32] recommend intraoperative wound irrigation with iodophor solutions such as PI before surgical closure, suggesting minimal harmful effects on tissues. Additionally, a randomized controlled trial reported that alcohol-based mouthwashes do not cause oral dryness [41]. Taken together, these findings indicate the need for further consideration and investigation into the use of PI for oral care in clinical practice.

This study had several limitations. First, although it was conducted as a randomized controlled trial, it was a preliminary study with a small number of participants, and it remains unclear whether the findings can be generalized. In addition, the study was not blinded, and the staff performing the interventions and collecting the samples were aware of the treatment group. Although significant reductions in bacterial counts were observed in the PI group, these findings should be interpreted with caution because the small sample size likely resulted in wide confidence intervals, indicating substantial uncertainty. Larger-scale studies will be necessary to provide more precise estimates of the effect size. This could introduce subconscious bias in how procedures were carried out or in sample handling. Second, the endpoint was not the incidence of VAP but rather the bacterial count above the endotracheal tube cuff. It is not yet known whether an increase in supra-cuff bacterial load directly leads to the development of VAP. However, since the incidence of VAP is relatively low and studying it as a primary endpoint would require a large sample size, we chose to use the bacterial count flowing into the lower respiratory tract as the endpoint for this preliminary study. This study was a preliminary investigation conducted with careful attention to patient safety, and no adverse events related to the interventions were observed. Based on our findings, we plan to conduct a larger-scale investigation to determine whether oral care using PI can reduce the incidence of VAP and improve patient outcomes.

## Conclusions

Before evaluating the effectiveness of oral care using PI for the prevention of VAP, we conducted a preliminary study with a small number of cases to examine the impact of PI application and toothbrushing on the bacterial count in secretions accumulating above the endotracheal tube cuff. The results showed that PI significantly reduced bacterial counts in supra-cuff secretions compared to toothbrushing in this preliminary study, and no adverse events were observed, indicating that the intervention was performed safely. These findings provide a basis for future larger-scale studies to evaluate the potential role of PI in oral care for intubated patients. In countries outside Japan, oral care with 0.12% CHX is widely used for intubated patients. Since the local oral administration of CHX is banned in Japan, our findings suggest that PI may serve as a useful alternative for oral care, as it has been shown to reduce bacterial load in supra-cuff secretions. However, this study was preliminary in nature and did not evaluate VAP incidence. Therefore, further large-scale studies are required to determine whether PI can contribute to the prevention of VAP.
